# A new genus of Alheitini (Lepidoptera, Mimallonidae, Lacosominae) from Central America

**DOI:** 10.3897/zookeys.1295.185731

**Published:** 2026-10-26

**Authors:** Ryan A. St Laurent, Daniel Herbin, Daniel Janzen, Winnie Hallwachs

**Affiliations:** 1 Museum of Natural History, University of Colorado Boulder, Boulder, CO, USA Museum of Natural History, University of Colorado Boulder Boulder United States of America; 2 Department of Ecology and Evolutionary Biology, University of Colorado Boulder, Boulder, CO, USA Department of Ecology and Evolutionary Biology, University of Colorado Boulder Boulder United States of America; 3 Department of Entomology, National Museum of Natural History, Smithsonian Institution, Washington, DC 20560-0127, USA Department of Entomology, National Museum of Natural History, Smithsonian Institution Washington, DC United States of America; 4 7, Le Clos de Lutché, 31380, Garidech, France Unaffiliated Garidech France; 5 Department of Biology, University of Pennsylvania, Philadelphia, PA 19104, USA Department of Biology, University of Pennsylvania Philadelphia United States of America

**Keywords:** Ebenaceae, *
Flammopenna
*, taxonomy

## Abstract

*
Flammopenna ***gen. nov**., is erected for *F. maya***sp. nov**., sole member of the new genus. This taxon had been recently included in a complete phylogenomic study of all named Mimallonidae genera but has eluded description until now. The new monotypic genus, recognizable by its relatively small size, falcate wing tips, and mostly uniform rusty-red coloration, occurs from Mexico throughout Central America to at least Costa Rica. Based on prior phylogenomic data, it is most closely related to a rarely collected South American genus, *Arianula* Herbin which contains two species distributed in the Amazon and Brazilian Cerrado. The new genus is so far the only mimallonid taxon known to feed on the plant family Ebenaceae.

## Introduction

Recent phylogenomic efforts to revise the higher classification of the historically poorly studied Mimallonidae, a family of moth endemic to the Americas, has resulted in nearly all described species being assigned to monophyletic, thoroughly diagnosed genera (St Laurent and Kawahara 2019; St Laurent et al. 2020). Two lineages in the latter study were not formally described, one in the lacosomine tribe Alheitini and another in the Mimalloninae. Here we describe the former, a member of Alheitini. In the past six years, observers on the popular community-science website iNaturalist (iNaturalist 2026) have regularly recorded this new genus and species from a wide range of localities, despite its rarity in collections from locations other than Costa Rica. This taxon, long known to the authors, therefore required formal description to ensure that this organism could be properly identified and recorded by the scientific and naturalist communities alike.

The Alheitini is one of three tribes of the Lacosominae, second most diverse subfamily of Mimallonidae after Cicinninae. Alheitini is the least broadly distributed tribe of lacosomines with only a handful of species reaching Central America, most being restricted to South America. Interestingly, the new genus presented here is the only genus of alheitines endemic to Mexico and Central America, and is the most northerly distributed member of the tribe.

## Materials and methods

We follow the methodology of our previous works on Mimallonidae taxonomy, dissections performed as in Lafontaine (2004) and morphological terminology and classification largely based on systematic revisions of the family from the past decade (St Laurent et al. 2018, 2020; St Laurent and Kawahara 2019). Examined material is derived from the following institutions:

**CDH** Coll. Daniel Herbin, Garidech, France;

**CMNH** Carnegie Museum of Natural History, Pittsburgh, Pennsylvania, USA;

**CNC** Canadian National Collection of Insects, Arachnids and Nematodes, Ottawa, Ontario, Canada;

**INHS** Illinois Natural History Survey Insect Collection, University of Illinois at Urbana-Champaign, Illinois, USA;

**MNCR** Museo Nacional de Costa Rica, San José, Costa Rica;

**MWM** Museum Witt, Munich, Germany;

**USNM** National Museum of Natural History [formerly United States National Museum], Washington D.C., USA;

**UVGC** Universidad del Valle de Guatemala, Arthropod collection, Guatemala City, Guatemala.

We also include records from iNaturalist.org, which when listed are followed by “observed by” and the iNaturalist user’s username in parentheses.

The figures were built using Adobe Photoshop Creative Cloud and the distribution map generated with SimpleMappr (Shorthouse 2010). When not explicitly given on labels, GPS coordinates for generating the map were inferred with Google Maps based on verbatim label data. For the holotype of the new species, labels are provided verbatim and separated by slashes.

## Results and discussion

### Flammopenna

Taxon classificationAnimalia

St Laurent & Herbin gen. nov.

9DB3AECF-BE95-548A-9A8B-8B8B2662566E

https://zoobank.org/32BB7440-C52A-451F-9EC8-3462B57720F9

Figs 1a–d, 2

#### Type species.

*Flammopenna maya* St Laurent and Herbin, sp. nov. by present designation.

#### Diagnosis.

**Figure 1.**
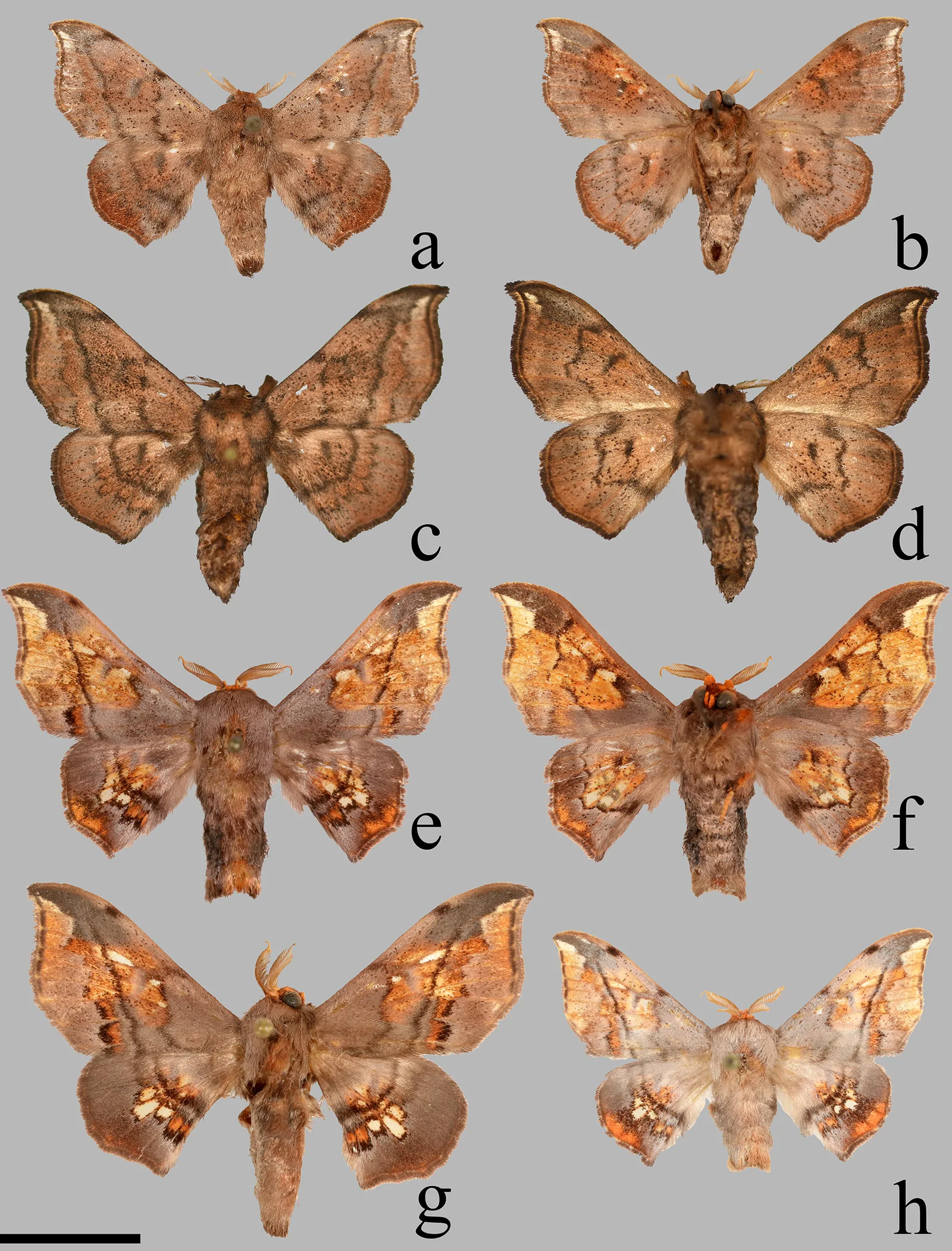
*
Flammopenna* and *Arianula* adult specimens. **a**. *F. maya* male holotype, Costa Rica, Guanacaste, 265 m (MNCR); **b**. As for (**a**) but ventral; **c**. *F. maya* female paratype, Costa Rica, Guanacaste, 05-SRNP-64183 (USNM); **d**. As for (**c**) but ventral; **e**. *A. haxairei* male, French Guiana, Piste Coralie (CDH); **f**. As for (**e**) but ventral; **g**. *A. haxairei* female, French Guiana, route nacional 2, piste de Belizon pk 6 (CDH); **h**. *A. feiranovensis* male, Brazil, Maranhão, Feira Novo do Maranhão, Retiro, 480 m (CDH). Scale bar: 1 cm.

Unmistakable among Alheitini or Lacosominae more broadly, appearing more similar to members of Cicinninae, such as *Cicinnus* Blanchard, 1852 or *Aceclostria* Vuillot, 1893, than to any other member of its tribe. The subdued, ruddy, earthen tones, poorly delimited antemedial, medial, and submarginal zones, and wavy antemedial and postmedial lines with a faint apical lunule are, in combination, not observed in any other Mimallonidae. Male genitalia are simple, particularly for Alheitini, best defined by the smoothly curving valvae, fist-like gnathos, and narrowly truncate uncus. Below the vinculum the anellus presents a pair of densely setose pads. The phallus is nondescript but very narrow, unlike the typically broad or otherwise highly modified phallus of other Alheitini, especially in comparison with that of its sister genus *Arianula* Herbin, 2012 (Fig. 1e–h), which bears a comparatively massive, complex hammerhead-like phallus (Fig. 3b, d). Likewise, female genitalia are notably simple for an alheitine, with only the setose, bilobed plate of the lamella postvaginalis being a noteworthy character. In both of our genitalia preparations the seemingly extremely fragile and thinly membraned corpus bursae was not recovered. Typically, however, mimallonid corpus bursae rarely contain complex features of diagnostic value and are usually globular or irregularly ovoid and bag-like.

#### Description.

**Figure 2.**
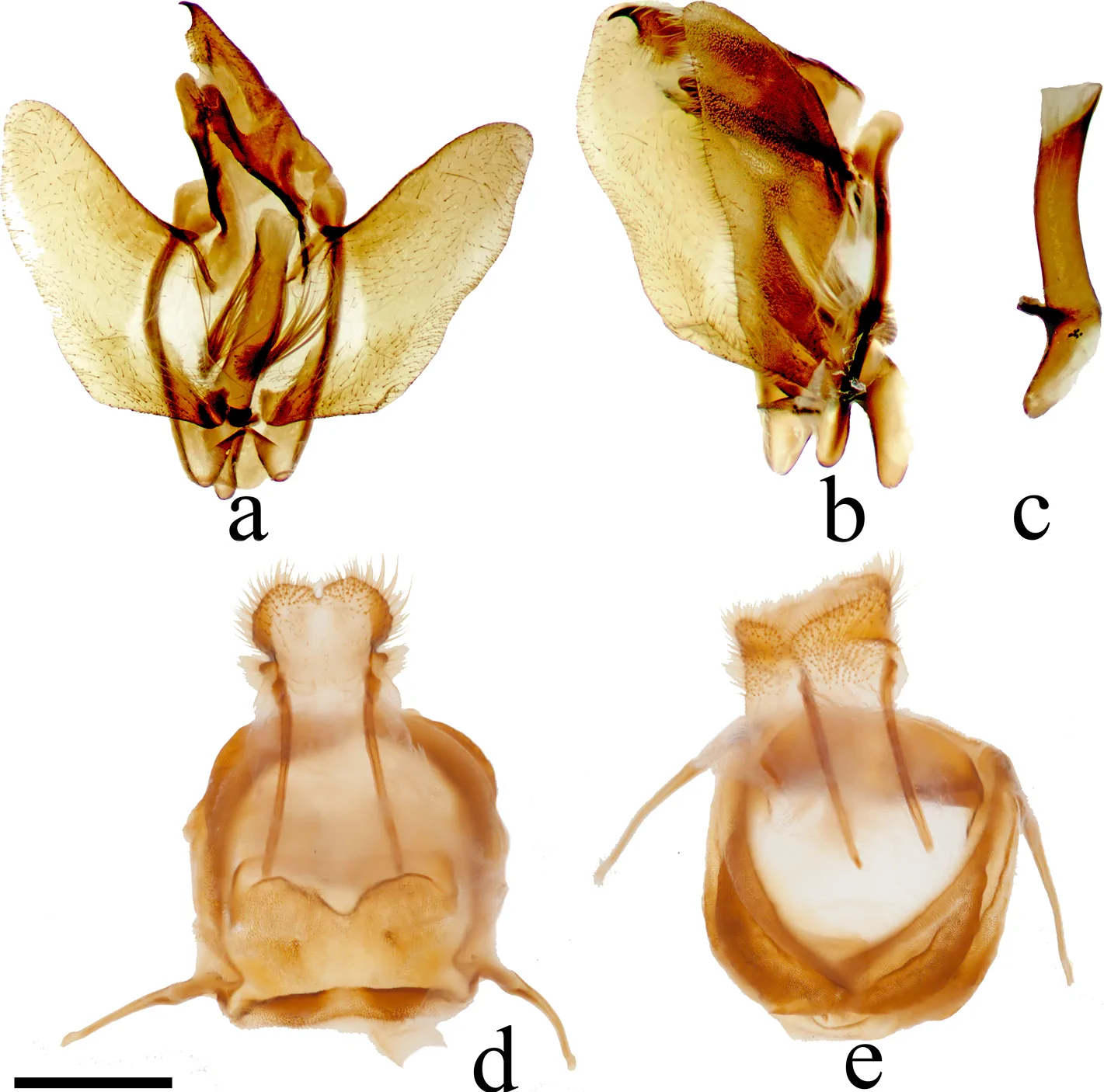
*
Flammopenna maya* type specimen genitalia. **a**. Male holotype, Costa Rica, Guanacaste, ventral with phallus attached, genitalia prep. D. Herbin ref H. 1791 (MNCR); **b**. As for (**a**) but lateral; **c**. As for (**a, b**) but phallus separated; **d**. Female paratype, Costa Rica, Guanacaste, ventral, St Laurent diss.: 4-28-25:1 (USNM); **e**. As for (**d**) but dorsal. Scale bar: 1 mm (**d, e**) [Scale bar applies only to female genitalia in d and e, male genitalia not to scale].

**Male. *Head***: frons pale tan-brown to dark brown; eyes very large, occupying more than two-thirds area of head as typical in Mimallonidae, bordered posteriorly by scales slightly darker than anterior margin of thorax; antenna coloration light tan, antenna almost entirely bipectinate, distalmost six or seven antennomers dentate; labial palpus 3-segmented with third segment very small, hardly noticeable beneath scales. ***Thorax***: coloration red-brown, densely scaled. ***Legs***: coloration as for thorax, vestiture thick. Tibial spurs short, covered in scales except for tip, less than half length of first tarsal segment. ***Forewing dorsum***: forewing length: 14.0–15.0 mm, avg.: 14.7 mm, *n* = 22. Triangular, deeply concave below apex convex along tornus. Ground color deep red-brown to paler rusty brown, with pale-gray undertone, overall sprinkled with dark-brown spatulate scales, antemedial, medial, and submarginal areas mostly concolorous but with faint gray apical lunule below darkened apex. Antemedial line present, wavy, dark brown; preapical postmedial line dark brown, ranging from well defined to very faint, apical portion may be interrupted or nearly absent. Discal mark present as dark to faint brown mark which may be outlined in gray. Fringe not contrasting with wing. ***Forewing ventrum***: as for dorsum but antemedial line absent, postmedial line wavier than on dorsum, discal spot more well defined. Retinaculum well developed. ***Hindwing dorsum***: coloration, patterning as for forewing dorsum, but antemedial line absent. May be more heavily adorned with spatulate scales. ***Hindwing ventrum***: following same pattern as forewing ventrum. Frenulum present as single bristle. ***Venation***: typical of Mimallonidae (venation preparation not made). ***Abdomen***: dorsal coloration as for thorax. Vestiture thick, long, somewhat denser at terminus. ***Genitalia***: (Fig. 2a–c) *n* = 2. Vinculum ovoid, when valvae spread base of vinculum pinches medially. Uncus simple, narrow, truncated distally. Gnathos well sclerotized, fist-like, somewhat flat, medially unfused. Valvae simple, smoothly curving, reaching nearly to apex of uncus. Diaphragm with paired patches of dense setae above phallus. Juxta fused to phallus, ventrally projected to connect to vinculum. Base of phallus weakly sclerotized, with poorly defined coecum phallus. Phallus cylindrical, curved upward distally, vesica simple. **Female. *Habitus***: as for male overall, and within the same range of coloration variation; wings somewhat broader and antennal pectinations shorter, dimorphism very limited, retinaculum absent, frenulum reduced and not visible but presumably as multiple bristles. Forewing length: 17 mm, avg.: 17 mm, *n* = 4. ***Female genitalia***: (Fig. 2d, e) *n* = 2. Tergite VIII narrowest mesally with wider sclerotized regions dorso-laterally. Apophyses anteriores slightly shorter than apophyses posteriores, much thicker, stouter, more curved. Lamella antevaginalis a narrow, sclerotized band, lamella postvaginalis a setose, more lightly sclerotized bilobed plate. Ductus bursae short and membranous, corpus bursae missing from both examined genitalia preparations. Papillae anales broadest mesally, appearing round viewed from terminus, overall densely covered in short setae.

**Figure 3.**
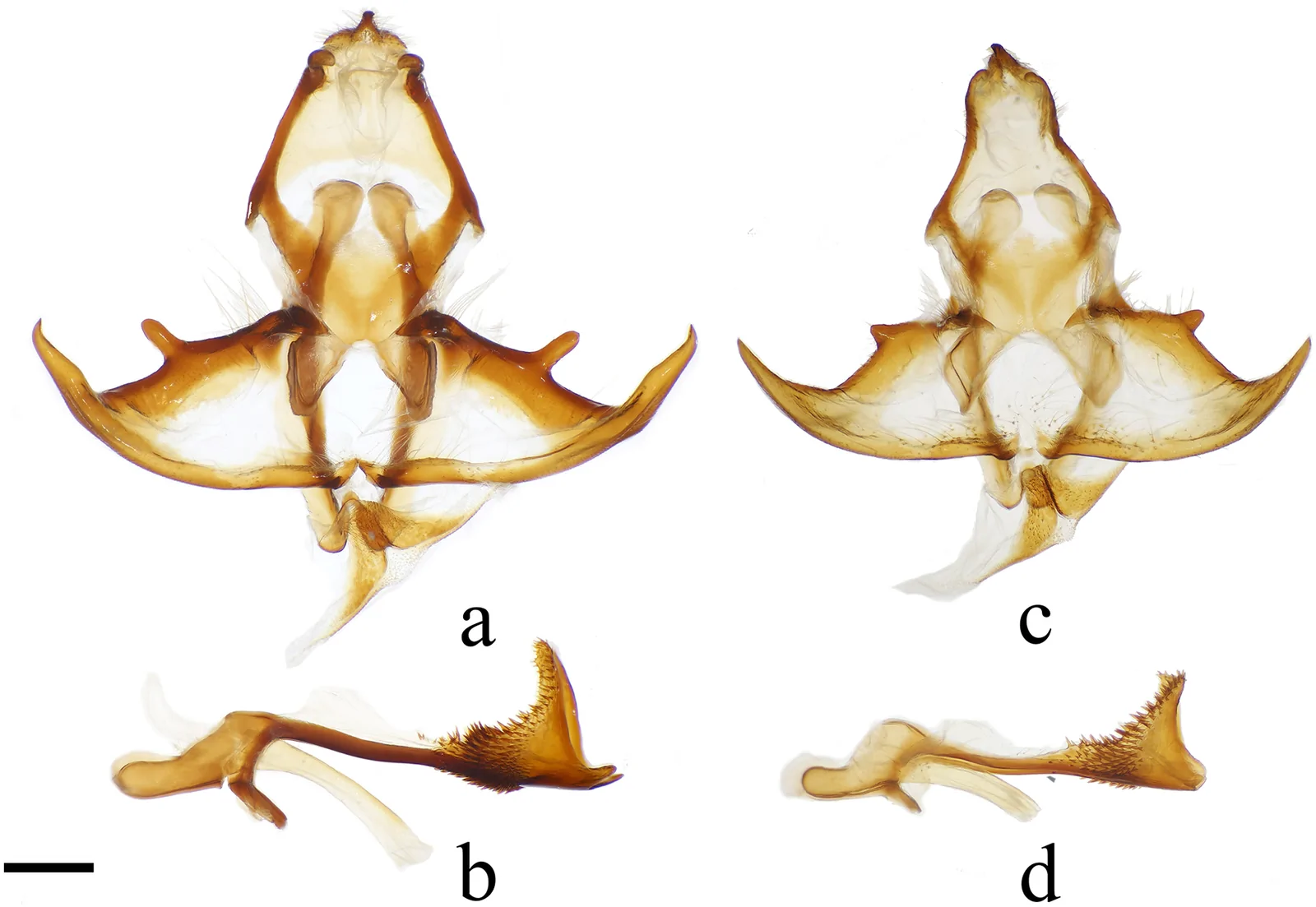
*
Arianula* male genitalia. **a**. *A. haxairei*, French Guiana, ventral, phallus excised, St Laurent diss: LEP50135 (MGCL); **b**. As for (**a**) but phallus; **c**. *A. feiranovensis*, Brazil, Distrito Federal, Estação Florestal, Cabeça do Veado, ventral, phallus excised St Laurent diss.: CNC001 (CNC); **d**. As for (**c**) but phallus. Scale bar: 1 mm.

#### Etymology.

*Flammopenna* is a feminine Latin construct of *flammeus*, meaning fiery red, and *penna*, feather (wing), referring to the rusty to fiery red wings.

#### Distribution.

**Figure 4.**
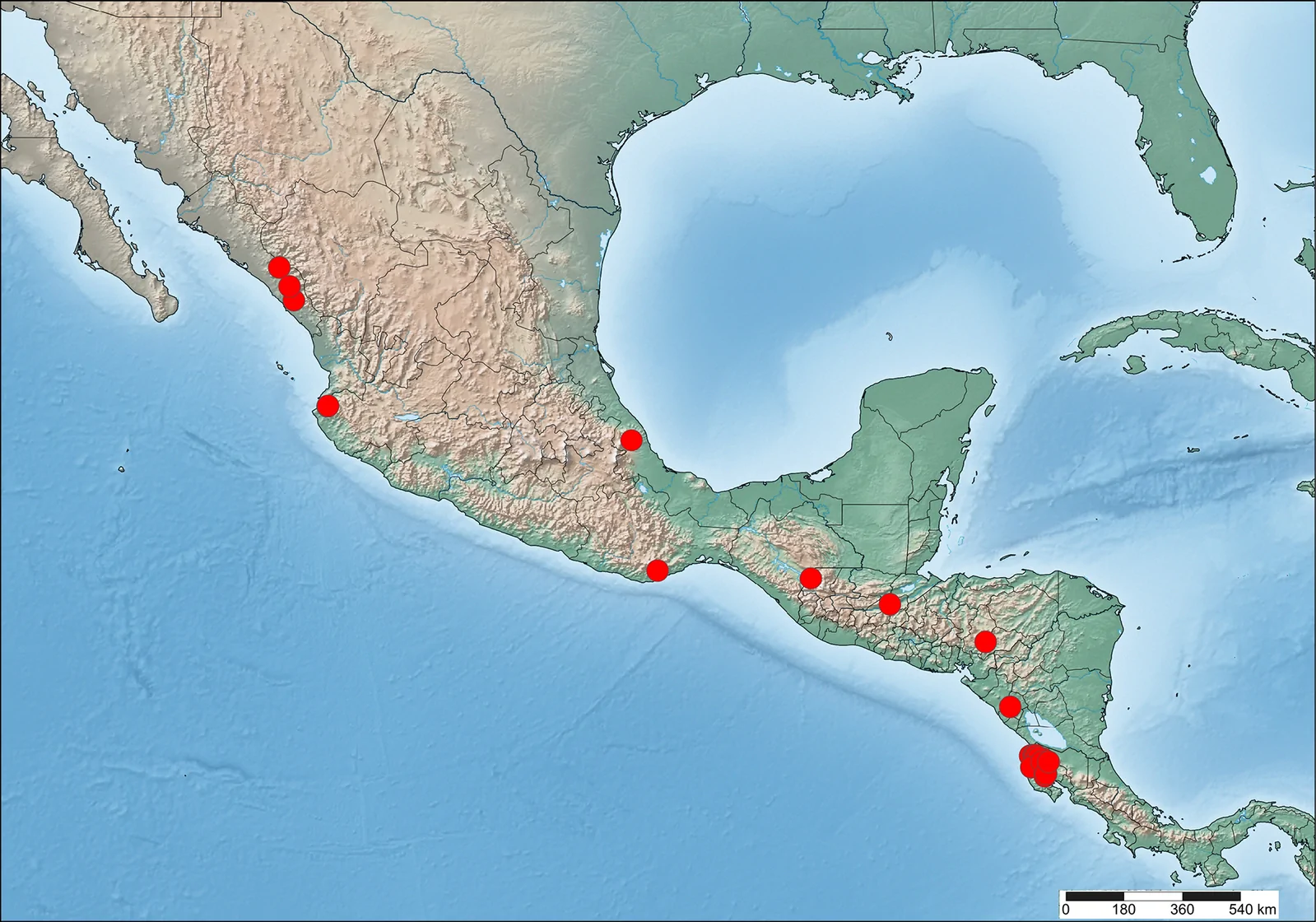
*
Flammopenna maya* locality records based on examined specimens and photographs from iNaturalist.org, note high density of sightings from Guanacaste, Costa Rica.

(Fig. 4) All records for the genus are from Central America, from as far north as Sinaloa, Mexico and on both Pacific and Atlantic coasts of Mexico, southward to northwestern Costa Rica. Records are spotty across this region, and its absence from most natural history collections suggests it is rarely seen (no records before the 1970s) and likely more widely distributed than our few records would suggest.

#### Natural history.

Apart from the habitats inferred from the collecting data reported herein, the only other natural history data available comes from Janzen and Hallwach’s (2017) rearing of this genus in Costa Rica. Based on several records, they report this genus to feed on *Diospyros acapulcensis* Kunth (Ebenaceae), a rather remarkable host plant for Mimallonidae, which are otherwise not known to feed on this family of plants (St Laurent et al. 2021). Janzen and Hallwach’s (2017) database also has a single record of the new genus from *Eugenia acapulcensis* Steudel (Myrtaceae) but interestingly we cannot find any other records to support this, and the same specific epithet as *Diospyros acapulcensis* is used, so it could be in error. Despite this, Myrtaceae is one of the most common host plant families used by Mimallonidae. No photos of the larvae are known.

#### Remarks.

While St Laurent et al. (2020) included this genus, unnamed, in their phylogenomic study of all known mimallonid genera, they did not choose to describe it at the time. *Flammopenna* was recovered with maximum support as sister to *Arianula* (Figs 1e–h, 3), a poorly known South American genus that is very dissimilar in external and genitalia morphology. *Flammopenna* was represented by a particularly long branch in that study, suggesting significant divergence from *Arianula*. A later study estimated the divergence time between *Arianula* and *Flammopenna* (as “New genus”) to be approximately 11.6 million years (St Laurent et al. 2021: supplemental fig. 2).

Currently, we attribute all specimens of *Flammopenna* to a single species, formally described below. However, the broad range, lack of continuous records across that range, and minor base pair differences in the mitochondrial “barcoding” gene, cytochrome c oxidase subunit I (COI) in publicly available molecular data, are at least suggestive that cryptic diversity exists in this genus (Hebert et al. 2003; Ratnasingham and Hebert 2007). Janzen and Hallwach’s (2017) rearing efforts in Costa Rica provide examples of two barcode clusters (Barcode Index Numbers, “BINs”) from the same sites but without morphological differences. Further collecting along its entire range and, ideally, population-level genomics would be useful to best understand the genetic diversity of the genus from Mexico to Central America.

### Flammopenna maya

Taxon classificationAnimalia

St Laurent & Herbin sp. nov.

298ECB4D-FB31-5412-8FA2-2710C3D015C8

https://zoobank.org/826F47CB-08B0-44E4-B056-984B66EC1E94

Figs 1a–d, 2

#### Type material.

***Holotype*. Costa Rica: Guanacaste**: • Parc National Santa Rosa, 265 m 19 Septembre 2009, 10°50'03.89"N, 85°36'47.68"W, Leg. D.Herbin/ ML Montagnani / HOLOTYPE ♂ *Flammopenna maya* St Laurent & Herbin, 2026 [red label]/ genitalia prep. D. Herbin ref H. 1791/ (MNCR).

***Paratypes***. (46 ♂, 8 ♀ total) **Mexico: Oaxaca**: • 1 ♂, 27 km 9° (NE) Huatulco, near Finca Monte-Carlo, 15°59.6'N, 96°06.3'W, 890 m, 26–31.VIII.2011, leg. V. Siniaev, O. Romanov, expedition Dr. R. Brechlin (MWM). **Guatemala: Zacapa**: • 1 ♂, Below San Lorenzo, 300 m nr. Pasabien Riv[er], 16–20.XI.1986, P. T. Dang/ MV Light Trap semi-desert, Acacia, Cactus & Euphorbia, 25–28 °C (CNC), 1 ♂, Sierra de Las Minas, Piste Santa Cruz à San Lorenzo, km10, 10.IX.2010, 575 m, 15°02'38.2"N, 89°40'07.5"W, leg. B. Vincent, genit. H1790 (CDH). **Huehuetenango**: • 1 ♂, Jacaltenango, La Laguna, 18.VI.2020, 15.77°, −91.853, Bc-Her 5699, leg. F. Camposeco (UVGC). **Nicaragua: Managua**: • 2 ♀, Laguna de Xiloa, 22.IX.1996 [1 ♀], 3–6.XI.1994 [1 ♀] (CMNH). **Costa Rica: Guanacaste**: • 1 ♂, Parc National Santa Rosa, 265 m 19.IX.2009, 10°50'03.89"N, 85°36'47.68"W, Leg. D.Herbin/ ML Montagnani, genit. ref H.859, Bc-Her3905 (CDH); • 3 ♂, Parc National Santa Rosa, 265 m 15.X.2010, 10°50'03.89"N, 85°36'47.68"W, leg. D.Herbin/ M.Laguerre (genit. H1562, H1791) (CDH); • 1 ♂, 2.3 km SE entrada de Parque Nacional Santa Rosa, 1.VI.1986, G. L. Godfrey (INHS). • 1 ♀, Las Ca[ñ]as, Rio Corobici, 26.VII.1967, O.S. Flint Jr., USNM-Mimal:2732 (USNM). 1 ♂, Sendero Mora, 480 m, 10.76828, −85.42567, emgd: 09/01/2005, coll: 08/10/2005, 05-SRNP-59293 (USNM). • 1 ♂, 1 ♀, Rio Azufrado, 95 m, 10.81224, −85.54438, emgd: 01/05/2006 [♂], 01/02/2006 [♀], coll: 12/12/2005 [♂], 12/12/2005 [♀], food plant: *Diospyros nicaraguensis*, 05-SRNP-64185 [♂], 05-SRNP-64183 [♀] (USNM). • 1 ♀, Vado Descanso, 70 m, 10.86336, −85.72535, emgd: 01/26/2006, coll: 12/28/2005, food plant: *Diospyros nicaraguensis*, 05-SRNP-64410 (USNM). • 5 ♂, La Perla (Tajo), 394 m, 10.76734, −85.43014, coll: 11/13/2007 [1 ♂], 12/09/2007 [1 ♂], 12/10/2007 [2 ♂], 06/10/2008 [1 ♂], light trap, 07-SRNP-113074, 111607, 1113427, 113426, 08-SRNP-102976 (USNM). • 5 ♂, Manta Potrero Grande, 20 m, 10.84337, −85.77975, coll: 05/22/2009 [1 ♂], 10/19/2009 [1 ♂], 12/15/2009 [1 ♂], 08/21/2009 [2 ♂], light trap, 09-SRNP-104310, 107808, 111268, 110091 (USNM). • 4 ♂, La Perla (Tanque), 366 m, 10.7676, −85.43243, coll: 12/09/2007 [1 ♂], 12/10/2007 [3 ♂], light trap, 07-SRNP-113664, 113665, 113666, 113332 (USNM). • 15 ♂, La Angostura, 300 m, 10.85592, −85.67017, coll: 05/25/2006 [2 ♂], 06/13/2007 [4 ♂], 06/25/2006 [4 ♂], 10/23/2006 [4 ♂], 09/26/2011 [1 ♂], light trap, 06-SRNP-104219, 105493, 105491, 104220, 105006, 105007, 105008, 108242, 108395, 105494, 105492, 108243, 105005, 108244, 11-SRNP-104369 (USNM). • 3 ♂, Sendero los Patos, 251 m, 10.82097, −85.63323, coll: 12/08/2007, light trap, 07-SRNP-112654, 112656, 112655 (USNM). • 1 ♀, Manta Calera, 342 m, 10.86576, −85.65112, coll: 08/31/2008, light trap, 08-SRNP-105677 (USNM). • 1 ♂, Plantacion Guachipelin, 161 m, 10.71349, −85.57117, coll: 10/28/2011, light trap, 11-SRNP-105464 (USNM). • 1 ♀, Mirador Saltillo, 141 m, 10.73137, −85.60075, light trap, 11-SRNP-105055 (USNM). • 2 ♂, 1 ♀, Luces, 575 m, 10.85362, −85.60942, coll: 05/31/2011 [1 ♂] 06/01/2011 [1 ♂, 1♀], light trap, 11-SRNP-103688 [St Laurent diss.: 4-28-25:2, ♀], 103535 [♂], 103689 [♂] (USNM).

#### Costa Rican specimens not included in type series.

• 1 ♂, 07-SRNP-111608 [no further data] (USNM). 1 ♂, 07-SRNP-111609 [no further data] (USNM). 1 ♂, 12-SRNP-13126 [no further data] (USNM). 1 ♀, 11-SRNP-13001 [no further data] (USNM). 1 ♀, Torre Esperanza, 85 m, 10.78938, −85.55098, emgd: 11/19/2005, coll: 10/23/2005, food plant: *Diospyros nicaraguensis*, 05-SRNP-62658, St Laurent diss.: 4-28-25:1[note that this and the following specimen belong to a different BIN from other Costa Rican material included in the type series] (USNM). • 1 ♀, La Angostura, 300 m, 10.85592, −85.67017, coll: 05/26/2006, light trap, 06-SRNP-104524 [note that this and the previous specimen belong to a different BIN from other Costa Rican material included in the type series] (USNM).

#### Online records (not collected).

(iNaturalist.org) **Mexico: Sinaloa**: • 1 ♀, Concordia, 23.48056, −106.20298, 6.XII.2023 (observed by mar_y_sierra_silvestre; https://www.inaturalist.org/observations/193257557). • 1 ♂, Pichilingue, 23.48059, −106.20309, 9.XI.2023 (observed by sosar and mar_y_sierra_silvestre; https://www.inaturalist.org/observations/190540586). • 1 ♂, Cosalá, 24.40261, −106.60728, 20.XI.2022 (observed by egamez; https://www.inaturalist.org/observations/146188651). • 1 ♂, Cosalá, 24.39846, −106.60556, 29.X.2022 (observed by egamez; https://www.inaturalist.org/observations/143476726). • 1 ♂, San Juan, 23.87822, −106.32290, 7.XI.2021 (observed by sergioescutia; https://www.inaturalist.org/observations/102338918). **Jalisco**: • 1 ♀, Puerto Vallarta, 20.54835, −105.27314, 22.VII.2023 (observed by troi_olivares; https://www.inaturalist.org/observations/174412447). • 1 ♂, Puerto Vallarta, 20.55902, −105.26026, 19.VII.2023 (observed by troi_olivares; https://www.inaturalist.org/observations/173822920). **Veracruz**: • 4 ♂, 1 ♀, Jilotepec, 19.60016, −96.84337, 27.V.2022, 26.VIII.2022, 31.VIII.2022, 21.IV.2023 (observed by carlosalbertoflores; such as https://www.inaturalist.org/observations/155855761). **Honduras: Francisco Morazán**: • 1 ♂, El Zamorano, 14.00698, −87.00759, 1.X.2025 (observed by ericvandenberghe; https://www.inaturalist.org/observations/318040466). **Costa Rica: Guanacaste**: • 1 ♂, Parque Nacional Palo Verde, 10.34514, −85.33868, 24.I.2025 (observed by pdar2 and nina64; https://www.inaturalist.org/observations/259678554). • 1 ♂, Sardinal, 10.52815, −85.74700, 23.XI.2024 (observed by mickeyratfink; https://www.inaturalist.org/observations/252709356). • 1 ♂, Liberia, 10.67756, −85.45525, 8.VI.2023 (observed by casseljs; https://www.inaturalist.org/observations/167947639). 1 ♂, Bagaces, 10.28004, −85.37115, VI.2022 (observed by lbmicheels; https://www.inaturalist.org/observations/153753661). • 1 ♀, Palo Verde Biological Station, 10.34529, −85.33899, 21.IX.2018 (observed by kueda; https://www.inaturalist.org/observations/17091749). • 1 ♂, Bagaces, 10.67676, −85.26679, 17.V.2025 (observed by lantinantoine; https://www.inaturalist.org/observations/286396410).

#### Diagnosis.

See genus.

#### Description.

See genus.

#### Etymology.

Maya refers to the Mayan civilization of Central America, from where this genus is best represented in Natural History Museums. Used as a noun in apposition.

## Supplementary Material

XML Treatment for Flammopenna

XML Treatment for Flammopenna maya

## Additional information

### Artificial Intelligence (AI) use

The authors accept full responsibility for the content of the manuscript, including the disclosure of any use of AI.

No AI tools were used in the preparation of this manuscript.

### Author ORCIDs

R.A. St Laurent https://orcid.org/0000-0001-6439-5249

D. Herbin https://orcid.org/0000-0002-1618-1606

D. Janzen https://orcid.org/0000-0002-7335-5107

W. Hallwachs https://orcid.org/0000-0002-5166-809X

### Data availability

All of the data that support the findings of this study are available in the main text.
